# Necrotizing Fasciitis of the Abdominal Wall Secondary to Complicated Appendicitis: A Case Report

**DOI:** 10.7759/cureus.39635

**Published:** 2023-05-29

**Authors:** Sirin Falconi, Christopher Wilhelm, Jocelin Loewen, Basem Soliman

**Affiliations:** 1 Surgery, Texas Tech University Health Sciences Center School of Medicine, Lubbock, USA; 2 General Surgery, Texas Tech University Health Sciences Center School of Medicine, Amarillo, USA; 3 Department of Surgery, Texas Tech University Health Sciences Center, Amarillo, USA

**Keywords:** abdominal wall infection, debridement. fascitis, necrotizing fascitis, enterocutaneous fisulae, necrotizing appendicitis

## Abstract

Acute appendicitis is one of the most common surgical emergencies worldwide. Many complications can occur secondary to complicated appendicitis including abscess formation, gangrene, sepsis, and perforation, rarely, leading to abdominal wall necrotizing fasciitis. The incidence of necrotizing fasciitis as a complication of ruptured appendicitis is extremely uncommon. The formation of an enterocutaneous fistula leading to this complication further emphasizes the rarity of such occurrence with few cases reported in the literature. Herein, we present a case of abdominal wall necrotizing fasciitis in a 72-year-old female presenting to the local emergency room with complaints of severe suprapubic abdominal pain associated with abdominal distension and acute onset foul-smelling drainage. Physical exam was significant for suprapubic and right lower quadrant abdominal tenderness with associated large indurated tender lesion and purulent weeping with large ecchymosis. Abdominal computed tomography (CT) revealed extensive subcutaneous emphysema, a large cavity with layering fluid extending into the peritoneal space, and a possible fistula formation between the intra-abdominal cavity and subcutaneous tissue. Following the diagnosis of probable necrotizing fasciitis secondary to fistula formation, the patient underwent emergent exploratory laparotomy and extensive debridement of necrotic tissue. In this report, we take the opportunity to highlight the importance of promptly recognizing and treating this uncommon complication and maintaining a high level of suspicion to prevent life-threatening consequences.

## Introduction

Acute appendicitis is one of the most common surgical emergencies worldwide with an estimated occurrence of 90-100 patients per 100000 per year [1,2]. Appendicitis is due to proximal luminal obstruction by a fecalith, lymphoid hyperplasia, or impacted stool [1]. CT scan of the abdomen and pelvis and transabdominal ultrasound are used to diagnose appendicitis [2]. Appendicitis is classified based on the severity of inflammation and divided into uncomplicated versus complicated [1,2]. Complicated appendicitis progresses to gangrene and/or perforation [1]. Once perforation occurs, the appendix contents leak into the peritoneal space, including bacteria, causing further inflammation involving the entire surrounding environment. This is a surgical emergency. Untreated peritoneal inflammation may extend into any structure in the vicinity and lead to catastrophic complications such as sepsis, abscess, or fistula formation [1,2]. An enterocutaneous fistula may extend into the abdominal wall allowing the spread of bacteria from the intraperitoneal cavity to the overlying abdominal fascia, muscle, subcutaneous fat, or skin [3]. Necrotizing fasciitis can ensue if one or more of its causative agents infects the fascia [4]. It is rapid and difficult to diagnose due to the lack of specific clinical features and skin changes in its initial phase [4]. For these reasons, mortality rates are still high. Prompt surgical debridement with antibiotic treatment and damage control surgery are life saving [4]. Damage control surgery focuses on the staged management of severely physiologically compromised patients that require surgical intervention [5]. 

Enterocutaneous fistula secondary to complicated appendicitis leading to necrotizing fasciitis is extremely rare. Thus, no guidelines are in place for the management of these cases. Here, we present a case of formation of an enterocutaneous fistula leading to necrotizing fasciitis of the abdominal wall in a 72-year-old female presenting with vague abdominal pain and foul-smelling abdominal drainage. This case highlights the importance of promptly recognizing and treating this uncommon complication and maintaining a high level of suspicion to prevent life-threatening consequences.

## Case presentation

**Figure 1:**
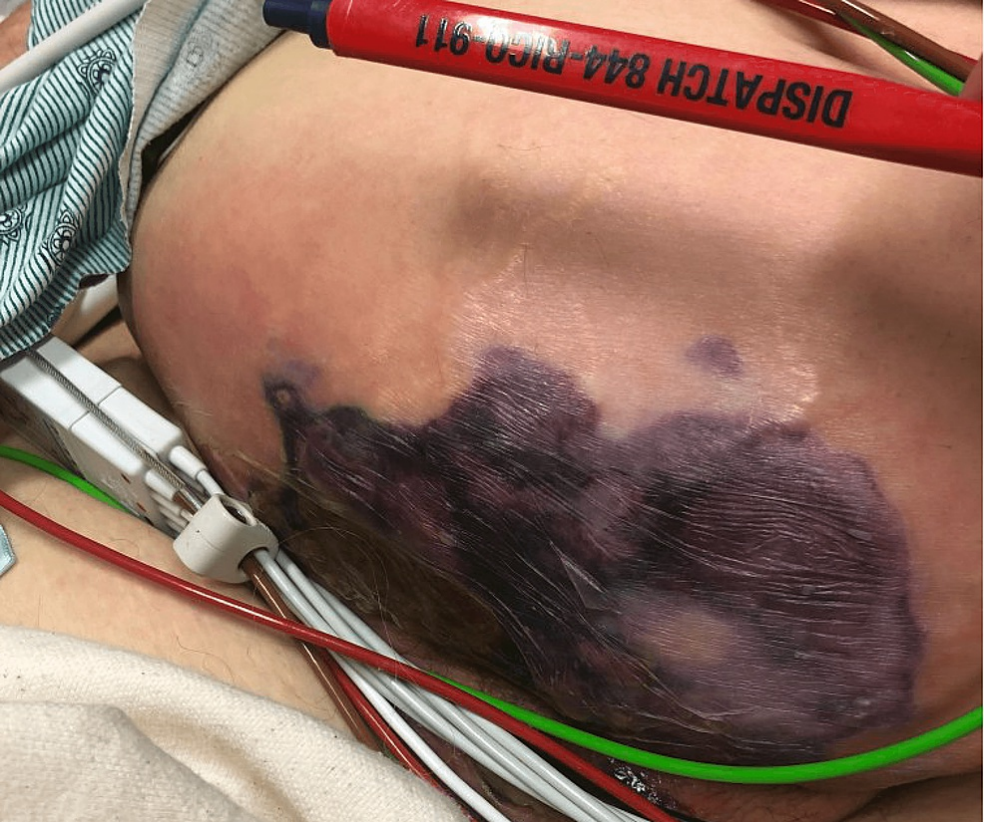
Large indurated tender lesion over 12 inches, purulent weeping with large ecchymosis, and extended edema and erythema

A 72-year-old female with a past medical history of prediabetes, arthritis, and methicillin-resistant Staphylococcus aureus (MRSA) positive abscess secondary to a recluse spider bite in 2005 presented to the local emergency department (ED) with complaints of severe suprapubic abdominal pain associated with abdominal distension and acute onset foul-smelling drainage. The patient reported first noting abdominal pain and discomfort one week prior to presentation. Symptoms progressively worsened culminating in blister formation and drainage (Figure 1). 

**Figure 2:**
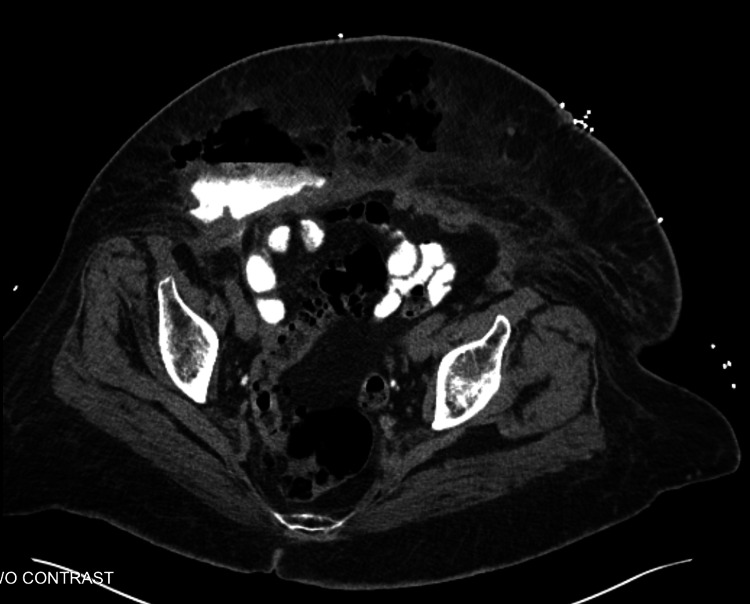
CT abdomen/pelvis with intravenous contrast showing extensive subcutaneous emphysema throughout the ventral abdominal/pelvic wall; a large cavity is seen within the subcutaneous tissues of the right lower quadrant with layering fluid and extravasation of oral contrast that appears to communicate with loops of small bowel in the right lower quadrant consistent with enteric fistula

In the ED, she was evaluated with laboratory and radiological studies. Laboratory data revealed elevated white blood count (WBC) at 28,000, elevated procalcitonin at 1.64, and elevated C-reactive protein (CRP) at 3.8. Vitals signs were within normal limits. Physical exam was significant for dry mucous membranes, suprapubic abdominal tenderness with associated large indurated tender lesion over 12 inches, purulent weeping with large ecchymosis, and extended edema and erythema. CT abdomen/pelvis with intravenous contrast showed extensive subcutaneous emphysema throughout the ventral abdominal/pelvic wall; a large cavity was seen within the subcutaneous tissues of the right lower quadrant, with layering fluid measuring on the order of 8 cm transverse by 5.2 cm anteroposterior by 11.6 cm craniocaudal extending into the peritoneal space. The fluid contained oral contrast and appeared to communicate with loops of small bowel in the right lower quadrant consistent with enteric fistula (Figure 2).

**Figure 3:**
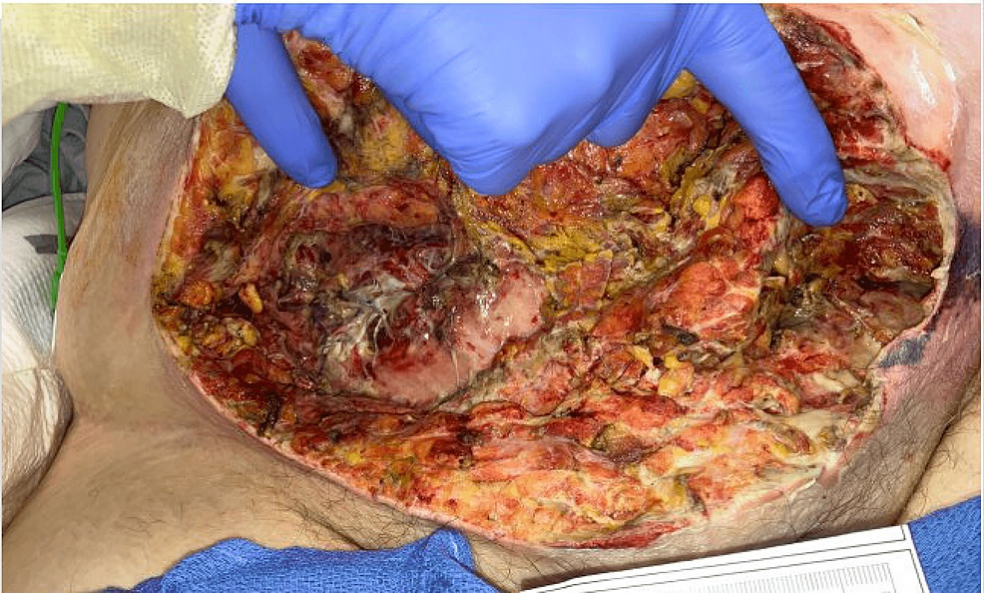
Abdomen following first irrigation and debridement of the necrotic tissue and contents

After obtaining wound cultures, the patient was started on broad-spectrum IV antibiotics with vancomycin and piperacillin-tazobactam, and general surgery referral was immediately obtained. A decision was made to proceed emergently to the operating room (OR) for exploratory laparotomy and debridement of possible necrotizing soft tissue infection. Sharp excisional debridement of ischemic skin and subcutaneous tissue was initiated with dissection down using electrocautery to debride all necrotic soft tissues involving the skin, subcutaneous tissues, fascia, and part of the muscle of the anterior abdominal wall in the lower abdomen and the suprapubic area (Figure 3).

There was evidence of stool leakage from the abdominal cavity through the abdominal wall with a visible enterocutaneous fistula. Upon exploration of the area of the enterocutaneous fistula, involvement of a ruptured appendix was identified. Ileocecectomy involving approximately 20 cm of the terminal ileum and cecum with primary ileocolic anastomosis was performed as well as enterocutaneous fistula resection. Post-operatively, wound cultures resulted positive for Escherichia coli, Aggregatibacter segnis, Bacteroides fragilis, and Clostridium perfringens. Following exploratory laparotomy, the patient was admitted to the intensive care unit (ICU) and returned to the operating room three more times for further irrigation and debridement of necrotic abdominal tissue and contents. The hospital course was complicated by bilateral pleural effusion development, electrolyte abnormalities, thrombocytopenia, and normocytic anemia. She remained on IV antibiotics throughout her hospital stay switching to ciprofloxacin and then cefepime. During the following weeks, the patient slowly deteriorated until succumbing to the complications of necrotizing fasciitis infection and dying. 

## Discussion

Necrotizing fasciitis is a rare subtle infection of the soft tissue that causes inflammation and necrosis of the fascia and consequently overlying muscle and skin [4]. There is no age or sex predilection for necrotizing fasciitis [6]. It occurs more frequently in diabetics, alcoholics, immunosuppressed patients, IV drug users, and patients with peripheral vascular disease [6]. Necrotizing fasciitis affects any region of the body, most commonly the abdomen, extremities, and perineum [6,7]. It is classified into two separate types, type I and type II, based on the bacteria involved. Type I is polymicrobial and involves non-group A streptococci plus anaerobes or facultative anaerobes. Type II involves group A beta-hemolytic streptococci alone or in combination with Staphylococcus [6]. A third type can be identified and is caused by vibrios [6]. The most common is type I [8]. Abdominal wall involvement results from postoperative complications of an incarcerated hernia, perforated viscus, appendicitis, and secondary enterocutaneous fistula [6,7]. 

Acute appendicitis is one of the most common surgical emergencies worldwide with a lifetime risk of 7%-8% [1,2]. Peak incidence is in the second or third decade of life [1]. The appendix is a finger-like outpouching of the cecum at the ileocecal junction that when obstructed, becomes inflamed. Once vascular supply is compromised, ischemia and gangrene occur weakening the wall and culminating in perforation. Many complications can occur following perforation including peritoneal inflammation leading to fistula formation. Trauma, malignancy, and bowel inflammation increase the risk of fistula formation [3]. When an enterocutaneous fistula forms between the appendix/colon/small bowel and abdominal wall, it becomes a bridge through which bacteria can travel. These bacteria include necrotizing fasciitis-causing bacteria such as group A Streptococcus, Staphylococcus aureus, Klebsiella, Clostridium perfringens, Escherichia coli, and anaerobic species [4]. An enterocutaneous fistula between the appendix and abdominal wall is a rare occurrence and is known as appendicocutaneous fistula [7].

Cases of necrotizing fasciitis secondary to a perforated appendix are rare [9]. With the addition of an enterocutaneous fistula formation, this case represents an extremely rare occurrence with few cases reported in the literature. A similar presentation was reported by Takeda et al. where a 76-year-old man presented with acute appendicitis leading to perforation into the retroperitoneum and formation of an enterocutaneous fistula, resulting in necrotizing fasciitis [7]. This case implemented prompt incision and drainage (I&D) followed by empiric antibiotic therapy [7]. Another example of ruptured appendicitis complicated by abdominal wall necrotizing fasciitis of unknown association was presented by Huang et al. where non-surgical conservative management was initially employed due to the patient’s immunocompromised state followed by fasciotomy and debridement due to increased complications [10].​ Despite extensive treatment, the patient died secondary to bacterial necrotizing fasciitis complicated by septic shock [10]. Suleimanov et al. reported a second case of necrotizing fasciitis of the abdominal wall secondary to perforated appendicitis where IV antibiotics were started immediately and the patient was taken to the OR for debridement, subsequently making a full recovery [11].

In our case, similarly to previous cases, the patient was started on immediate intravenous broad-spectrum antibiotic therapy with piperacillin/tazobactam and vancomycin due to the atypical presentation and high level of suspicion for necrotizing fasciitis; she was quickly taken to the OR for exploratory laparotomy and I&D of abscess and debridement. Although prompt surgical treatment was started and the patient was taken to the OR multiple times for further debridement, she succumbed to the complications of necrotizing fasciitis infection and died several weeks later. 

Immediate management with prompt surgical debridement and broad-spectrum antibiotic therapy is considered the standard treatment for necrotizing fasciitis due to the devastating damage caused by this disease process [6-11]. Of those who received treatment within 24 hours, the mortality rate was found to be 12.5% versus 72.7% in those where treatment was delayed by four days [9]. It is recommended to start broad-spectrum antibiotics prior to culture and sensitivity results to prevent further dissemination of the disease [9]. Finally, complete excision of the non-viable tissues is the only cure for necrotizing fasciitis [8]. Other adjuvant treatment options include hyperbaric oxygen therapy and vacuum-assisted closure therapy which have shown some improvement but are not considered the standard of care and should not delay surgical debridement [8]. 

Necrotizing fasciitis is extremely difficult to treat, especially with delayed diagnosis. Its presentation as a complication of ruptured appendicitis is uncommon with mortality rates up to 30% [10,11]. As mentioned previously, an immunosuppressed state is a risk factor for necrotizing fasciitis, putting the elderly population at a higher risk of such a complication. Furthermore, the rate of complicated appendicitis appears to also be higher in this population for similar reasoning such as decreased immune response and the presence of multiple comorbidities [2]. Elderly patients appear to present with milder symptomatology and possible misclassification [2]. Therefore, a high level of suspicion is necessary in this population group. 

## Conclusions

Necrotizing fasciitis is a disease process with significant mortality rates even with appropriate treatment. Its occurrence as a complication of ruptured appendicitis leading to enterocutaneous fistula formation is extremely uncommon with few cases reported in the literature. Although such complication is rare, a high level of suspicion is necessary to prevent life-threatening consequences. Immediate management with prompt surgical debridement and broad-spectrum antibiotic therapy is imperative to prevent further damage. Complete excision of the non-viable tissues is the only cure for this deadly infection. 
